# Is FH a curable disease?

**DOI:** 10.21542/gcsp.2026.39

**Published:** 2026-08-31

**Authors:** Nabil G. Seidah

**Affiliations:** Montreal Clinical Research Institute (IRCM), affiliated to the University of Montreal, Laboratory of Biochemical Neuroendocrinology, Montreal, Quebec H2W 1R7, Canada

**The history of familial hypercholesterolemia (FH) represents one of the most remarkable examples of translational medicine, progressing from a clinical observation to molecular genetics and, ultimately, to highly effective targeted therapies.**

The story began in 1938 when Carl Müller first described the association between tendon xanthomas, markedly elevated serum cholesterol, premature myocardial infarction, and autosomal dominant inheritance^1^. Remarkably, he recognized the hereditary nature of the disorder decades before the discovery of the LDL receptor (LDLR) and proposed that FH represented an inborn error of metabolism. These pioneering observations laid the foundation for the modern understanding of FH^1,2^.

Between 1938 and 1975, before the introduction of statins, treatment options were limited to low-cholesterol diets, bile acid sequestrants (often poorly tolerated because of gastrointestinal adverse effects), nicotinic acid, and fibrates. These therapies generally reduced LDL cholesterol (LDLc) by less than 20%, leaving patients with homozygous FH (HoFH) at extremely high cardiovascular risk. Individuals with biallelic LDLR-null mutations frequently died before 30 years of age from premature myocardial infarction^3^. The introduction of plasmapheresis in the mid-1970s represented a major advance, transiently lowering LDLc by 50–70%^3,4^, although the procedure remained invasive and required lifelong repeated treatments.

A breakthrough occurred in 1974–1975 with the discovery of the LDL receptor (LDLR) and the demonstration that loss-of-function variants cause severe hypercholesterolemia^5^. LDLR was subsequently established as the principal disease-causing gene in FH, accounting for approximately 80–90% of HoFH cases^6^. This discovery directly led to the development of statins, orally active inhibitors of HMG-CoA reductase, the rate-limiting enzyme in cholesterol biosynthesis. By reducing intracellular cholesterol synthesis, statins upregulate hepatic LDLR expression and typically lower LDLc by 25–40%^7^. However, because their efficacy depends on residual LDLR activity, statins are markedly less effective in patients carrying biallelic LDLR-null mutations^5,8^.

The next major therapeutic advance was ezetimibe, the first orally active inhibitor of intestinal cholesterol absorption. By selectively blocking the NPC1L1 transporter in enterocytes, ezetimibe decreases dietary and biliary cholesterol absorption, reduces hepatic cholesterol stores, and induces compensatory upregulation of hepatic LDLRs^9^. When added to statins, ezetimibe provides an additional 15–25% reduction in LDLc. Like statins, however, its efficacy depends on functional LDLRs and is therefore substantially diminished in patients with receptor-negative HoFH^8^.

Another transformative milestone occurred in 2003 with the discovery of PCSK9^10^, a central regulator of LDLR degradation and cholesterol homeostasis^11–13^. This discovery rapidly led to the development of PCSK9-targeted therapies^14^, including monoclonal antibodies (evolocumab and alirocumab), small interfering RNA (inclisiran), and, more recently, orally active inhibitors and genome-editing approaches. In patients with residual LDLR activity, these therapies, particularly when combined with statins, can reduce LDLc by approximately 50–70% and have dramatically improved cardiovascular outcomes^14^.

In contrast, patients with biallelic LDLR-null HoFH require therapies that lower LDLc independently of LDLR function. Currently, these include lomitapide, an inhibitor of microsomal triglyceride transfer protein (MTP); evinacumab, a monoclonal antibody targeting angiopoietin-like protein 3 (ANGPTL3); and LDL apheresis. By comparison, LDLR-dependent therapies—including statins, ezetimibe, and PCSK9 inhibitors—provide only limited benefit in this patient population.

## Conclusions and future perspectives

The evolution of FH therapy over the past 85 years illustrates the transition from nonspecific lipid-lowering strategies to precision molecular medicine. The discovery of LDLR established the molecular basis of FH, while the identification of PCSK9 revolutionized lipid-lowering therapy and substantially reduced cardiovascular risk for millions of patients.

Today, the greatest unmet clinical need remains the treatment of patients with biallelic LDLR-null HoFH. LDLR-independent strategies—including ANGPTL3 inhibition, ApoC3 inhibition, and emerging gene-editing technologies such as siRNA and CRISPR-based approaches—represent the most promising therapeutic direction. In particular, the combination of ANGPTL3 and ApoC3 inhibition has strong mechanistic appeal because it simultaneously targets lipoprotein production and clearance through LDLR-independent pathways^15^. Although this approach remains investigational, it offers considerable promise for patients with receptor-negative HoFH.

After decades in which treatment was largely palliative, advances in precision therapeutics have fundamentally transformed the outlook for FH. While a universally effective cure has not yet been achieved, the convergence of LDLR-independent therapies with gene replacement and gene-editing technologies provides unprecedented optimism that curative treatments may eventually become a clinical reality.

## Commentary on the current study

The case report by Mahmoud Barbir and colleagues provides a remarkable illustration of how the management of HoFH has evolved over the past 35 years. In 1990, a 33-year-old woman with biallelic LDLR-null HoFH presented with advanced atherosclerotic cardiovascular disease, severe coronary artery disease, aortic valve stenosis, heart failure, and an LDLc concentration of 13 mmol/L despite treatment with statins, bile acid sequestrants, nicotinic acid, probucol, and intermittent LDL apheresis.

She subsequently underwent combined orthotopic heart and liver transplantation, resulting in an immediate reduction of LDLc to 2.1 mmol/L. This dramatic improvement was maintained for decades with relatively low-dose immunosuppressive therapy. Although long-term treatment was complicated by progressive renal failure requiring permanent hemodialysis, this exceptional intervention enabled survival for more than 35 years despite an otherwise devastating disease.

Historically, before the advent of LDL apheresis and modern lipid-lowering therapies, untreated patients with biallelic LDLR-null HoFH frequently experienced myocardial infarction during childhood or adolescence and rarely survived beyond early adulthood. Although contemporary LDLR-independent therapies have substantially improved survival, receptor-negative HoFH remains the most challenging form of FH to treat.

Despite major advances in lipid-lowering therapy, no currently available pharmacological regimen consistently achieves optimal LDLc reduction in patients with near-complete absence of LDLR activity. Among existing therapies, ANGPTL3 inhibition remains the only clinically validated LDLR-independent strategy capable of producing robust LDLc lowering in receptor-negative HoFH^16–18^.

If this patient was treated today, liver transplantation from a healthy donor would restore hepatic LDLR function, thereby enabling effective treatment with LDLR-dependent therapies such as statins, ezetimibe, and PCSK9 inhibitors. Whether this combination would ultimately constitute a true cure remains an open question.

Looking ahead, several emerging therapies—including ANGPTL3 RNA interference (RNAi), ApoC3 inhibition using siRNA or antisense oligonucleotides, LDLR gene replacement, CRISPR-mediated correction of LDLR mutations, hepatocyte transplantation, and stem cell-derived liver therapies—offer realistic prospects for further improving outcomes in these patients.

Finally, the physicians who cared for this patient over more than three decades deserve special recognition. Their extraordinary clinical commitment transformed what was once an invariably fatal disease into one associated with prolonged survival and an improved quality of life, highlighting both the remarkable progress achieved in FH treatment and the challenges that remain.
